# Bone turnover is adequately suppressed in osteoporotic patients treated with bisphosphonates in daily practice

**DOI:** 10.1186/1471-2474-12-167

**Published:** 2011-07-21

**Authors:** Danielle A Eekman, Irene EM Bultink, Annemieke C Heijboer, Ben AC Dijkmans, Willem F Lems

**Affiliations:** 1 Department of Rheumatology, VU University Medical Center, Amsterdam Netherlands; 2 Department of Clinical Chemistry, VU University Medical Center, Amsterdam Netherlands; 3 Department of Rheumatology, Jan van Breemen Institute, Amsterdam, Netherlands

## Abstract

**Background:**

Monitoring osteoporosis therapy by measurement of bone turnover markers (BTMs) might detect non-compliance in an earlier stage of anti-osteoporosis treatment and improve persistence.

**Methods:**

BTMs were measured in two groups. The first group consisted of patients newly diagnosed with osteoporosis and starting treatment. We observed which proportion of patients had a decrease of serum levels of procollagen type 1 N-terminal propeptide (P1NP) and C-terminal crosslinking telopeptide (CTX) greater than the least significant change (LSC) after 3 months of treatment. Secondly, we determined which proportion of patients who were treated with bisphosphonates for ≥ 3 months reached the biological goal of therapy, BTMs in the lower half of the normal premenopausal range. P1NP and CTX were also measured in a reference population of 34 healthy premenopausal women.

**Results:**

In the first group 31 patients were included, in 25 patients (81%) levels of both markers decreased with ≥ LSC, in the other patients a possible explanation was found.

In the second group 95 patients were included, in 95% the serum P1NP levels and CTX levels were in the lower half of the premenopausal range. In 6 of the 7 patients with a level above the premenopausal range a possible explanation was found.

**Conclusion:**

A decrease in bone turnover ≥ LSC can be observed in the majority of newly treated patients. In chronically treated patients, 95% have a bone turnover in the premenopausal range. In most patients with inadequate suppression of BTMs during bisphosphonate treatment, an explanation was found. Monitoring treatment effect with BTMs in daily practice is feasible, and might be an additive tool in improving therapy compliance.

## Background

Osteoporosis is a major public health problem that primarily affects elderly women. Reduced bone mineral density increases fracture risk and results in considerable morbidity, mortality, and health care costs [1]. In clinical studies treatment with oral bisphosphonates has been shown effective, reducing vertebral fracture risk with 40-50% [2,3]. An important problem in daily clinical practice however, is the poor long-term compliance and persistence, diminishing the intended effect on BMD and fracture risk [4,5].

Although repeated dual × ray absorptiometry (DXA) measurements are sometimes used to monitor therapy, changes in bone mineral density (BMD) are small over the years and reliable repeated BMD measurements are only possible after 1-2 years [6,7].

The least significant change (LSC, the difference that represents a statistically significant change) of BTMs can already be detected after 3-6 months of treatment [8] with anti-resorptives and enables earlier monitoring of therapy. The size of the LSC varies between different types of bone markers and depends on the intra-individual variation and coefficient of variation of the assay [9]. The goal of treatment has been determined as a reduction of serum BTM levels within the premenopausal range or to decrease serum BTM levels with a percentage greater than the LSC [10].

It has been suggested that reinforcing patients by informing them about the change in their BTMs during treatment might have beneficial effects on persistence to therapy and even on reducing the risk of vertebral fractures. In the Improving Measurements of Persistence on Actonel Treatment (IMPACT) study osteoporotic patients treated with risedronate were divided into two groups, one group receiving reinforcement based on changes in BTMs (a decrease of > 30%, no change, an increase > 30%) and one group receiving no reinforcement. Patients with positive reinforcement (> 30% decrease in BTM) showed a significant improvement in persistence and had a lower incidence of new radiologically reported vertebral fractures. Patients receiving negative reinforcement however (> 30% increase in BTM), showed a significant decline in persistence compared to the group receiving no reinforcement [11].

The aim of this study is to observe in daily clinical practice in which proportion of patients starting with bisphosphonate treatment the serum levels of procollagen type 1 N-terminal propeptide (P1NP, marker of bone formation) and C-terminal crosslinking telopeptide (CTX, marker of bone resorption) show a decrease ≥ LSC, three months after starting with therapy. Additionally, we observed in which proportion of patients, diagnosed with osteoporosis and treated for at least three months with bisphosphonates, the serum level of P1NP and CTX is in the lower half of the premenopausal range.

## Methods

To observe short- and long-term effects we included two different types of patients visiting the osteoporosis outpatient clinic at the VU University Medical Center. Group 1 consisted of patients newly diagnosed with osteoporosis and starting treatment with anti-resorptive therapy. In group 2 patients were included who were already diagnosed with osteoporosis and treated with bisphosphonates for at least three months. Patients were included as part of a study previously approved by the ethics committee at the VU University Medical Center.

In both groups height and weight were measured, and body mass index (BMI) was calculated. DXA measurements (Hologic 4500, Waltham, Mass., USA) of the total hip and spine (L1-L4) were performed before start of treatment. Instant Vertebral Assessment (IVA) or a spine radiograph were performed to detect possible vertebral fractures. Treatment was started in case of: a T score of ≤ -2.5 in either hip and/or spine, a T score of ≤ -2 and a clinical fracture, a radiologically confirmed vertebral fracture, or the use of prednisone in a dose of at least 7.5 mg/day during at least three months.

All patients were screened for secondary causes of osteoporosis through laboratory testing before start of treatment, including erythrocyte sedimentation rate, thyroid stimulating hormone, free T4, creatinine, urea, alkaline phosphatase, calcium and 25(OH) vitamin D.

The first choice of treatment was an oral bisphosphonate. In case of (gastro-intestinal) contraindications patients were given zoledronic acid intravenously once yearly.

In group 1 P1NP and CTX serum levels were measured before start of treatment and at their follow up visit three months later in the same assay. During this visit patients were asked about compliance and the occurrence of side effects, in which case another bisphosphonate was prescribed.

In group 2 one cross-sectional measurement of serum P1NP and CTX levels was performed and patients were asked about compliance during their visit to the outpatient clinic.

Blood samples for both markers were withdrawn in fasting state, early in the morning. P1NP was measured by radio immunoassay (Orion Diagnostica, Espoo, Finland) and CTX by electrochemiluminescence (Roche Diagnostics, Mannheim, Germany).

Patients with a recent fracture (less than 6 months before the outpatient clinic visit) were excluded.

### Reference population

In order to determine the premenopausal range of fasting serum levels of BTMs, P1NP and CTX serum levels were also measured in a group of 34 healthy premenopausal women born between 1968 and 1989.

### Least significant change in P1NP and CTX

The LSC was calculated with the formula: Z × √2 × √ (AV² + IV²), where AV represents the analytical change in our laboratory, IV the intra-individual variation and Z is the Z-score. At the 0.95 confidence level, Z is 1.96 [12]. The AV for CTX was 3% and for P1NP 8%. We used an IV of 10%, based on results found in earlier studies [9,13]. Using these values for IV and AV the calculated LSC for CTX was 29% and for P1NP 36%.

### Statistics

Changes over time of serum P1NP and CTX levels were analyzed using a paired samples t test. Differences between groups were analyzed using an independent t test. The software used for statistical analyses was SPSS for Windows, version 15 (SPSS, Chicago, IL).

## Results

Because the values were not distributed normally in the reference population, we calculated the median (range) P1NP level (45 (28-110) μg/L) and CTX level (405 (115-905) ng/L). These ranges are comparable to those found in earlier studies [14,15].

Baseline characteristics are presented in Table 1. In group 1 thirty-one patients that subsequently visited the osteoporosis outpatient clinic were included, over two thirds of whom were women. Thirteen patients were diagnosed with osteoporosis based on a T-score of ≤ -2.5 in their spine and/or hip and sixteen were osteopenic. Two patients had a normal BMD, however, they both had a vertebral fracture.

**Table 1:** Baseline characteristics

Variables		Group 1 n = 31	Group 2 n = 95
Age (years)	mean (SD)	66 (9)	67 (11)
Gender (female)	n (%)	22 (71)	75 (79)
BMI (kg/m2)	mean (SD)	26 (5)	25 (5)
T score spine	mean (SD)	-2.2 (1.1)	-2.2 (1.3)
T score hip	mean (SD)	-1.6 (0.8)	-1.6 (0.9)

Baseline characteristics of group 1 (patients starting treatment n = 31) and group 2 (patients treated > 3 months n = 95).

BMI = body mass index

Bisphosphonate treatment consisted of alendronate (70 mg weekly) in 15 patients, 5 risedronate (35 mg weekly), 4 ibandronate (150 mg monthly) and 7 zoledronic acid (5 mg intravenously once yearly) because of gastrointestinal contraindications for oral bisphosphonates.

After a median treatment duration of 4 months, the levels of P1NP and CTX decreased both significantly with 50% and 63% respectively (p < 0.001, Table 2). Eighty-one percent of the patients showed a decrease greater than the LSC of both markers. Nearly all patients reached premenopausal CTX and P1NP values (respectively 94 and 97%, Table 3).

**Table 2:** Changes in BTM levels

Variables		Baseline	Follow up	Decrease %
P1NP (μg/L)	mean (range)	47 (15-93)	22 (9-49)	50 *
CTX (ng/L)	mean (range)	363 (177-718)	128 (10-446)	63 *

Significant changes in BTM levels in group 1 (n = 31) during treatment with bisphosphonates.

* p < 0.001

P1NP = procollagen type 1 N-terminal propeptide; CTX = C-terminal crosslinking telopeptide

**Table 3:** Distribution of Bone Marker Values Compared to Premenopausal Reference Range Before and On Bisphosphonate Therapy (at least 3 months)

	P1NP (μg/L)		CTX (ng/L)	
	**Baseline**	**Follow up**	**Baseline**	**Follow up**

Below reference range (%)	4	71	0	52
Lower half of reference range (%)	14	26	65	45
Upper half of reference range (%)	42	3	35	3
Above reference range (%)	0	0	0	0

P1NP = procollagen type 1 N-terminal propeptide; CTX = C-terminal crosslinking telopeptide

In Table 4 values of CTX and P1NP, type of treatment and possible explanation in the patients that did not decrease ≥ LSC are summarized.

**Table 4:** Possible explanations in patients with a decrease ≤ LSC

Patient	CTX baseline (ng/L)	CTX follow up (ng/L)	P1NP baseline (μg/L)	P1NP follow up (μg/L)	Decrease CTX (%)	Decrease P1NP (%)	Possible explanation	Bisphosphonate
1	365	150	35	34	59	3 *	Recent fracture §	Alen
2	250	13	15	11	95	27 *	Prednisone use	Alen
3	209	52	25	20	75	20 *	Alcohol abuse	Alen
4	429	446	54	30	+4 *	44	Paraproteinemia	Iban
5	177	151	85	40	15 *	53	Non-compliance	Ris
6	311	365	45	49	+17 *	+9 *	Non-compliance	Alen

Values of CTX and P1NP, type of bisphosphonate and possible explanation in patients that did not decrease ≥ LSC during treatment with a bisphosphonate in the first few months.

* a decrease of CTX or P1NP < LSC

§ during the follow up visit the patient mentioned pain in his foot which turned out to be a recent fracture of a metatarsal bone.

Alen = alendronate 70 mg once weekly, Iban = ibandronate 150 mg once monthly, Ris = risedronate 35 mg once weekly

P1NP = procollagen type 1 N-terminal propeptide; CTX = C-terminal crosslinking telopeptide; LSC = least significant change

At the outpatient clinic visit after three months patients were asked about their therapy. One patient indicated not to have taken the medication at all (nr 6 in Table 4), another patient had taken the medication less frequently than prescribed and/or occasionally during a meal (nr 5). There were no differences between patients that did show a decrease of ≥ LSC and those that did not, in age, BMI, vitamin D level, BMD and type of bisphosphonate.

In group 2 ninety-five patients were included, of whom 31 patients had also been included in group 1. In this subgroup of 31 patients, the second measurement of CTX and P1NP was used for analysis. Group 2 consisted mainly of women. Twenty-four had a T-score of ≤ -2.5 in their spine and/or hip, 47 had a T-score in the osteopenic range, while 19 patients had a normal BMD. The patients with a normal BMD were either using prednisone chronically (3) or had one or more vertebral fractures (16). Of 5 patients no recent DXA measurement (within 6 months of the cross-sectional measurement of CTX and P1NP) was available.

Bisphosphonates used were: 40 alendronate (70 mg weekly), 24 risedronate (35 mg weekly), 18 zoledronic acid (5 mg intravenously, once yearly), 6 ibandronate (150 mg monthly), 6 pamidronate (60 mg intravenously every three months) and 1 (alendronate 70 mg/cholecalciferol 2800 IU weekly). Three patients were non-compliant, among whom nr 5 and 6 described in Table 4. The mean serum levels of P1NP (22 μg/L) and CTX (137 ng/L) were in the lower half of the premenopausal range. Of all patients 90 (95%) had a serum P1NP level in the lower half of the premenopausal range. Serum CTX levels were measured in 84 patients, of whom 80 (95%) had a level in the lower half of the premenopausal range.

## Discussion

In our study, performed in a clinical setting, levels of P1NP and CTX decreased significantly in most patients during the first months of treatment with bisphosphonates. And, importantly, almost all reached the biological target of therapy; the lower half of the premenopausal range. Moreover, in 95% of the patients chronically treated with bisphosphonates bone turnover markers were within the aimed premenopausal range. Because significant positive effects on bone turnover can be observed even 3 months after starting therapy, an adequate suppression of serum P1NP and CTX serum levels might be a valuable tool to monitor early treatment effect and persistence to therapy.

Our present study is the first demonstrating a rapid and significant decrease of BTMs in the first months of treatment with bisphosphonates and a reduction of BTMs to premenopausal levels in almost all osteoporotic patients on chronic bisphosphonate treatment in a daily clinical practice setting. The results of the present study are supported by previous studies performed in the setting of clinical trials. A group of 693 women diagnosed with osteoporosis and at least one vertebral deformity were treated with risedronate 5 mg daily. Their urinary CTX decreased with 60% after 3-6 months of treatment [16]. In a smaller group of 67 patients treated with monthly ibandronate, median serum CTX level was reduced with 70%, already three days after therapy initiation [17]. In a recent publication from the HORIZON trial P1NP serum levels decreased with a median of 56% and β-CTX serum levels decreased with 51% after three yearly infusions with zoledronic acid [18].

The fact that almost all patients in our study showed a similar decrease is quite remarkable considering the fact that patients participating in a clinical trial are usually more compliant. Although our study was not designed to monitor or influence compliance, patients were aware that the effect of therapy would be monitored, possibly this information did have a positive effect on persistence.

The suggestion that therapy compliance might be improved by communicating the monitoring of BTMs with the patients treated, is supported by the results of the IMPACT study. The IMPACT study described earlier was designed to investigate the effect of such early reinforcement using BTMs in patients treated with risedronate. Depending on their BTM response (> 30% increase, -30% to 30% change, > 30% decrease) patients received the message that their response was, respectively, poor, stable or good [11].

According to the criteria of the IMPACT trial, no patient in our study had a poor response and six patients in group 1 had a stable response of one, or both markers. In three patients P1NP levels remained stable while CTX decreased ≥ LSC. A possible explanation for this discrepancy might be the fact that the decrease in bone formation (represented by P1NP) was lagging behind the decrease in bone resorption (represented by CTX), a normal phenomena in the treatment with anti-resorptives. The suppression of bone formation can occur up to three months later than the suppression of resorption [8]. In one patient both markers remained stable, which was explained by non-compliance.

In the group of patients using chronic bisphosphonates, most patients had a CTX and P1NP level below the premenopausal median. In two patients both markers were above the median. One of the two was non-compliant, the other patient was also diagnosed with rheumatoid arthritis, which can cause elevated BTMs [19]. In two patients only CTX was above the median, one of whom was being treated with prednisone for polymyositis and phenytoin for epilepsy. Of the two patients with a P1NP level above the median, one was using a wheelchair (decreased mobility) and the other was non-compliant.

Our study has several limitations. Although the group patients with a cross-sectional measurement is quite substantial, the group of patients starting bisphosphonates is relatively small. To our knowledge however, no previous studies concerning the follow up of BTMs in daily clinical practice have been published before. Secondly, we did not measure compliance in a standardized manner. In our study population self-reported compliance rates were quite high. More objective information from digital dispensers or pharmacies possibly could have revealed lower rates. Most patients however had a decline of BTMs during follow up and/or levels in the desired therapeutic range when measured cross-sectionally, suggesting that they were indeed taking their medication as prescribed. In both groups a number of patients (7 and 18) were treated with zoledronic acid intravenously, obviously compliance is not an issue in these patients. Thirdly, over half of the patients starting treatment with a bisphosphonate already had BTMs in the premenopausal range. (Table 3) However, in most of these patients a decrease of > 30% could be seen, Finally, the measurement of markers of bone turnover in itself has several restrictions, of which the analytical and pre-analytical variability are the most important. The former depends on the marker and the method. Factors that influence pre-analytical variability can be divided into two categories; controllable factors, such as diet, diurnal variation, physical exercise. And variability that can not be controlled for, such as age, gender, menopausal status, diseases characterised by changes in bone turnover, and medications [8]. In order to minimize the effect of both forms of variability all samples were drawn in fasting state, and results were compared to the results of measurements in healthy premenopausal controls, using the same method. By establishing a cut-off value (LSC, which takes both intra- and intervariability of the test into account) above which the change is significant, the effect of variability is further minimized. Ideally separate reference groups for different populations would be created (e.g. men, or patients with RA).

## Conclusion

In conclusion, monitoring anti-resorptive treatment using BTMs in clinical practice is feasible and might be an important tool in monitoring early treatment effect and compliance.

## Competing interests

The authors declare that they have no competing interests.

## Authors' contributions

DE collected data, drafted the manuscript and performed statistical analysis. IB participated in the design of the study and helped to draft the manuscript. AH helped to draft the manuscript and performed the laboratory tests. BD helped to draft the manuscript. WL conceived of the study, participated in its design and coordination and helped to draft the manuscript. All authors read and approved the final manuscript.

## Pre-publication history

The pre-publication history for this paper can be accessed here:

http://www.biomedcentral.com/1471-2474/12/167/prepub

## References

[B1] Sambrook P Cooper C Osteoporosis Lancet 2006 367 2010 2018 10.1016/S0140-6736(06)68891-0 16782492

[B2] Cranney A Tugwell P Adachi J Weaver B Zytaruk N Papaioannou A Meta-analyses of therapies for postmenopausal osteoporosis. III. Meta-analysis of risedronate for the treatment of postmenopausal osteoporosis Endocr Rev 2002 23 517 523 10.1210/er.2001-3002 12202466

[B3] Cranney A Wells G Willan A Griffith L Zytaruk N Robinson V Meta-analyses of therapies for postmenopausal osteoporosis. II. Meta-analysis of alendronate for the treatment of postmenopausal women Endocr Rev 2002 23 508 516 10.1210/er.2001-2002 12202465

[B4] Siris ES Selby PL Saag KG Borgstrom F Herings RM Silverman SL Impact of osteoporosis treatment adherence on fracture rates in North America and Europe Am J Med 2009 122 S3 13 19187810 10.1016/j.amjmed.2008.12.002

[B5] Yood RA Emani S Reed JI Lewis BE Charpentier M Lydick E Compliance with pharmacologic therapy for osteoporosis Osteoporos Int 2003 14 965 968 10.1007/s00198-003-1502-4 14504697

[B6] Lenchik L Kiebzak GM Blunt BA What is the role of serial bone mineral density measurements in patient management? J Clin Densitom 2002 5 Suppl S29 S38 12464709 10.1385/jcd:5:3s:s29

[B7] Lodder MC Lems WF Ader HJ Marthinsen AE van Coeverden SC Lips P Reproducibility of bone mineral density measurement in daily practice Ann Rheum Dis 2004 63 285 289 10.1136/ard.2002.005678 14962964 PMC1754906

[B8] Szulc P Delmas PD Biochemical markers of bone turnover: potential use in the investigation and management of postmenopausal osteoporosis Osteoporos Int 2008 19 1683 1704 10.1007/s00198-008-0660-9 18629570

[B9] Baim S Miller PD Assessing the clinical utility of serum CTX in postmenopausal osteoporosis and its use in predicting risk of osteonecrosis of the jaw J Bone Miner Res 2009 24 561 574 10.1359/jbmr.090203 19257812

[B10] Bergmann P Body JJ Boonen S Boutsen Y Devogelaer JP Goemaere S Evidence-based guidelines for the use of biochemical markers of bone turnover in the selection and monitoring of bisphosphonate treatment in osteoporosis: a consensus document of the Belgian Bone Club Int J Clin Pract 2009 63 19 26 19125989 10.1111/j.1742-1241.2008.01911.x PMC2705815

[B11] Delmas PD Vrijens B Eastell R Roux C Pols HA Ringe JD Effect of monitoring bone turnover markers on persistence with risedronate treatment of postmenopausal osteoporosis J Clin Endocrinol Metab 2007 92 1296 1304 10.1210/jc.2006-1526 17244788

[B12] Srivastava AK Vliet EL Lewiecki EM Maricic M Abdelmalek A Gluck O Clinical use of serum and urine bone markers in the management of osteoporosis Curr Med Res Opin 2005 21 1015 1026 10.1185/030079905X49635 16004668

[B13] Scariano JK Garry PJ Montoya GD Wilson JM Baumgartner RN Critical differences in the serial measurement of three biochemical markers of bone turnover in the sera of pre- and postmenopausal women Clin Biochem 2001 34 639 644 10.1016/S0009-9120(01)00273-9 11849624

[B14] Glover SJ Gall M Schoenborn-Kellenberger O Wagener M Garnero P Boonen S Establishing a reference interval for bone turnover markers in 637 healthy, young, premenopausal women from the United Kingdom, France, Belgium, and the United States J Bone Miner Res 2009 24 389 3997 10.1359/jbmr.080703 18665786

[B15] de Papp AE Bone HG Caulfield MP Kagan R Buinewicz A Chen E A cross-sectional study of bone turnover markers in healthy premenopausal women Bone 2007 40 1222 1230 10.1016/j.bone.2007.01.008 17331821

[B16] Eastell R Barton I Hannon RA Chines A Garnero P Delmas PD Relationship of early changes in bone resorption to the reduction in fracture risk with risedronate J Bone Miner Res 2003 18 1051 1056 10.1359/jbmr.2003.18.6.1051 12817758

[B17] Binkley N Silverman SL Simonelli C Santiago N Kohles JD Dasic G Monthly ibandronate suppresses serum CTX-I within 3 days and maintains a monthly fluctuating pattern of suppression Osteoporos Int 2009 20 1595 1601 10.1007/s00198-008-0827-4 19145396

[B18] Delmas PD Munoz F Black DM Cosman F Boonen S Watts NB Effects of yearly zoledronic acid 5 mg on bone turnover markers and relation of PINP with fracture reduction in postmenopausal women with osteoporosis J Bone Miner Res 2009 24 1544 1551 10.1359/jbmr.090310 19338427

[B19] Garnero P Landewe R Boers M Verhoeven A Van Der LS Christgau S Association of baseline levels of markers of bone and cartilage degradation with long-term progression of joint damage in patients with early rheumatoid arthritis: the COBRA study Arthritis Rheum 2002 46 2847 2856 10.1002/art.10616 12428224

